# Malmö Treatment Referral and Intervention Study—High 12-Month Retention Rates in Patients Referred from Syringe Exchange to Methadone or Buprenorphine/Naloxone Treatment

**DOI:** 10.3389/fpsyt.2017.00161

**Published:** 2017-08-31

**Authors:** Martin Bråbäck, Lars Ekström, Katja Troberg, Suzan Nilsson, Pernilla Isendahl, Louise Brådvik, Anders Håkansson

**Affiliations:** ^1^Addiction Center Malmö, Division of Psychiatry, Malmö, Sweden; ^2^Faculty of Medicine, Department of Clinical Sciences, Psychiatry, Lund University, Lund, Sweden; ^3^Department of Clinical Pathology, Sahlgrenska University Hospital, Gothenburg, Sweden; ^4^Department of Infectious Diseases, University Hospital Skåne, Malmö, Sweden

**Keywords:** opioid dependence, syringe exchange, methadone, buprenorphine, maintenance treatment

## Abstract

**Background:**

Heroin dependence is associated with high mortality. Opioid agonist treatment (OAT) with methadone or buprenorphine has strong evidence for treatment of this relapsing condition. In our setting, OAT has been associated with strict and demanding intake procedures, often with requirements of social stability, but also high, approximately 80 percent 12-month retention rates. In a recent randomized controlled trial, we demonstrated high rates of successful rapid referral from a syringe exchange programme (SEP) to treatment with methadone or buprenorphine, including actual treatment initiation. The objectives of this study were to assess 12-month retention rates, in order to assess whether a novel referral program of current drug users at a SEP would achieve retention rates comparable to more traditional intake procedures.

**Methods:**

The present report is a 12-month follow-up of 71 patients who successfully started treatment with methadone or buprenorphine/naloxone. Patient data from baseline and at 12 months were collected.

**Results:**

Out of the 71 patients who started treatment, 58 (82%) were still in treatment after 12 months.

**Conclusion:**

This was a population, referred from a SEP, with a high drug use severity on admission and no pretreatment requirement for social stability, but there were still high retention rates at 12 months comparable to regular opioid agonist clinics in our setting.

## Introduction

Heroin dependence is a chronic relapsing disorder with high mortality (1, 2). In several clinical trials, opioid agonist treatment (OAT) with methadone or buprenorphine have been shown to reduce mortality and also to be efficient with regard to reduced drug use criminal activity, HIV risk behavior, and to suppress symptoms of the addictive disorder itself (3–13). Retention in treatment is critical and the benefits of remaining in OAT for at least 1 year is well established (14–17). The mean 12-month retention is approximately 50–60% (17–22).

Patients at syringe exchange programme (SEP) may seem a rational target population for recruitment to methadone and buprenorphine treatment programs, but successful recruitment in this setting may be a challenge. Individuals attending a SEP report a high degree of drug use severity and a high prevalence of psychiatric comorbidity compared to other out-of-treatment or treatment seeking opioid users (23–27). There is also evidence that when referred to OAT, SEP participants have a poorer treatment response (28). In contrast, in the recently published Malmö Treatment Referral and Intervention Study (MATRIS), we showed that SEP can be efficient for rapidly transferring heroin-dependent patients to evidence-based treatment with methadone and buprenorphine with high rates of referral (99%) and treatment entry (96%) (29). Previous data from the present setting, traditionally characterized by less rapid and high-threshold intake procedures, have demonstrated high rates of treatment retention for patients included in methadone or buprenorphine treatment (30).

In the present study, we aimed to study whether the rapid intake procedure—without the requirement for social stability in a population with a high degree of substance-related problems—could still be associated with high 12-month retention rates.

## Materials and Methods

### Study Design

The present study is a 12-month follow-up of the patients who started treatment at an outpatient clinic for OAT is a result of our two-group randomized controlled trial MATRIS. Inclusion took place at the SEP in Malmö, Sweden, and all participants were referred to the same research treatment facility run by Malmö Addiction Center. Inclusion for the study took place between October 21, 2011 and April 1, 2013, and all participants signed informed consent. MATRIS was designed to test the effectiveness of a SEP for referring patients to treatment with methadone or buprenorphine but also to assess the effect of a strength-based case management intervention (CMI) with regard to treatment entry (31–33). The participants were randomized to a CMI (intervention group) or to a referral-only condition (control group). Regardless of intervention type, we saw high referral rates with 71 patients successfully starting maintenance treatment. The present study is a follow-up to 12 months with regard to retention in treatment. The study was approved by the regional ethics board Lund, Sweden and registered at www.clinicaltrials.gov with nr NCT01457872.

### Setting

This study took place in Malmö, which is situated in the region of Skåne in the southern part of Sweden. Skåne has a population of around 1.3 million and the biggest city is Malmö with a population of approximately 300,000. SEPs were established in two Swedish cities (Malmö and Lund) in the 1980s and in addition to exchange of syringes and needles, the programs offer interventions aimed at motivating the individual to accept care and treatment. The SEP in Malmö is run by the Department of Infectious Diseases. In Sweden, methadone, as treatment for opioid dependence was introduced as early as the late 1960s and buprenorphine in 1999. OAT is only allowed at special addiction treatment units, the thresholds for treatment have been high and availability low resulting in waiting lists at many treatment facilities. The retention rates for patients admitted to maintenance treatment have, however, traditionally been high in Sweden (30).

### Participants and Recruitment

Participants were individuals who had been enrolled in OAT with buprenorphine/naloxone or methadone through the MATRIS study. Eligible participants for that study were opioid-dependent individuals enrolled in the SEP. About 1,000 individuals were actively participating in the SEP during the time of the study and about half of them stated their drug of choice to be heroin. Eligible participants were initially approached by the SEP staff and if interested in participating within days scheduled for formal study inclusion and baseline interview, which was conducted by two social workers trained in case management. Exclusion criteria were pregnancy, severe unstable psychiatric condition, inability to understand information, and to provide informed consent, and if the patient was already formally participating in treatment. At the baseline interview, the participants were asked about medical, psychiatric, and drug use history. After finishing the baseline interview, the participants were randomized. Both groups also received an appointment to the physician at the outpatient clinic for OAT 7 days after the randomization. At the medical examination, at the outpatient clinic, the participants provided a urine sample for urine toxicology and the physician assessed whether the participants were eligible for OAT with methadone or buprenorphine according to national regulations. OAT was started 4 days after the medical examination if the participant was deemed eligible. The choice of medication was based on clinical characteristics and outside of the study protocol. The methods for this part of the study are described in detail elsewhere (29). Out of the 75 recruited and randomized patients, 71 started treatment with methadone or buprenorphine. The patients who entered treatment had a mean age of 39 with a range from 23 to 65. Of the participants, 52 (73%) were males and 19 (27%) females. Thirty-one per cent reported that they had their own apartment and 9% were employed. The patients were allowed to state multiple primary sources of income and the most common were social welfare (61%), crime (55%), and from family or partner (39%). On average, they started using heroin at age 21 and reported injecting, 21 of 30 days prior to study inclusion. Eighty per cent reported hepatitis C infection, 70% reported previous defined overdoses, and 31% previous suicide attempts. On average, they rated their quality of life on a visual analog scale 32 of 100.

The patients received standard care at the outpatient clinic for OAT and were followed up with regard to retention in treatment.

### Statistical Analysis

Survival analysis was conducted. The number of days in treatment was used as the time-dependent variable. The outcome variable was treatment retention at 12 months. The statistical analysis was made with IBM SPSS Statistics (version 22).

## Results

**Figure 1:**
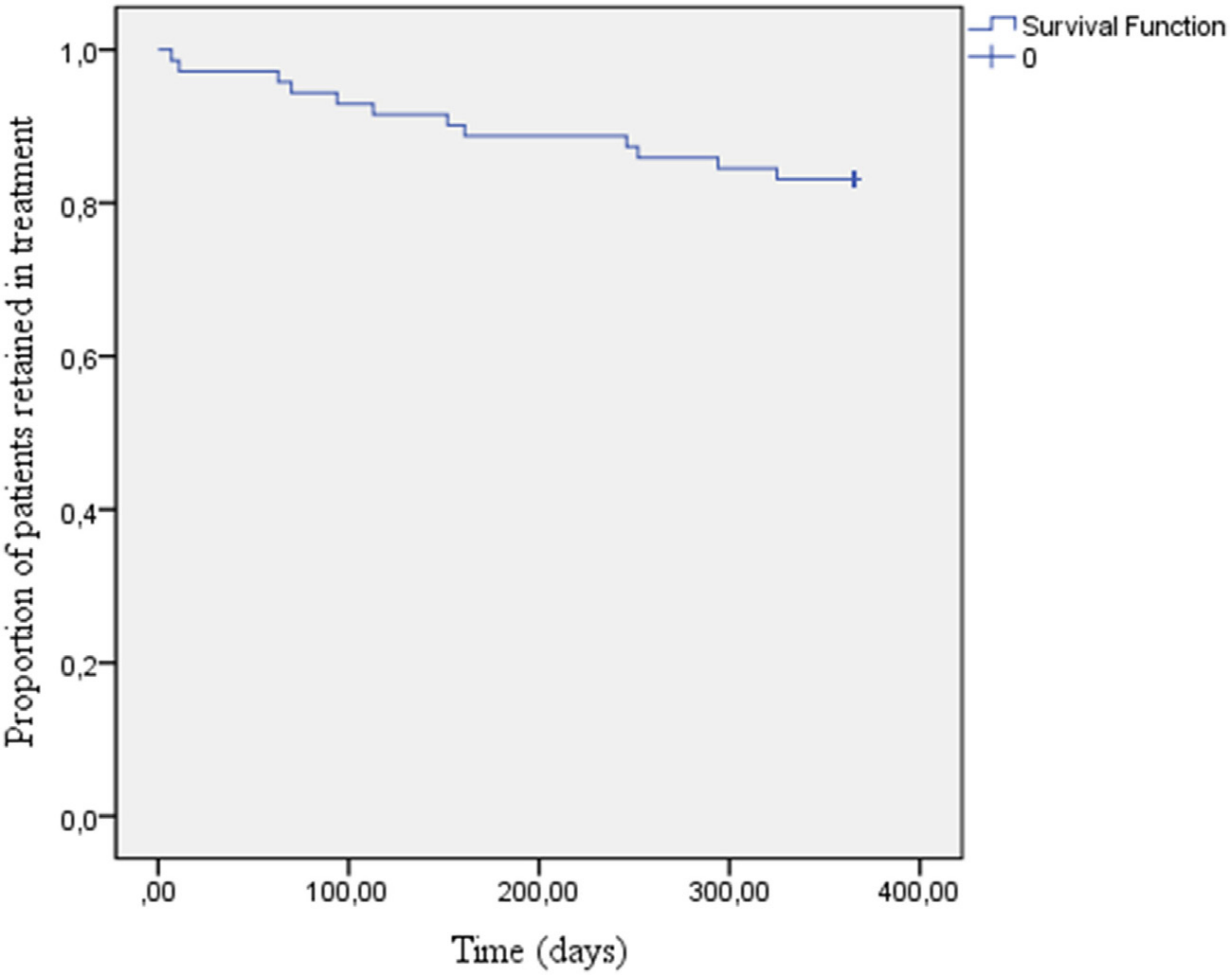
Patients retained in treatment over time.

Out of the 71 patients who entered treatment, 67 (94%) were still in treatment after 3 months, 63 (89%) after 6 months, and 58 (82%) after 12 months (Figure 1).

## Discussion

In a previous publication, our group showed that SEPs can be efficient for structured referral of opioid-dependent individuals to evidence-based treatment (29). In this 12-month follow-up of the patients who entered treatment, we demonstrate high-retention rates despite having a study population with a high degree of substance-related problems at baseline, and despite using a rapid referral procedure from an out-of-treatment SEP setting.

Studies examining retention rates when it comes to SEP referrals are scarce, and to our knowledge, this is the first one outside the US. Neufeld et al. reported that SEP referrals in Baltimore were less likely to complete 1 year in treatment (35%), compared to other referrals (56%). Referral condition was, however, not associated with outcome when it was adjusted for baseline variables known to be associated with poorer response to OAT, such as high drug use severity, injection drug use, and unemployment (28, 34, 35). The average 12-month retention overall, not for subjects specifically referred from SEPs, have in different studies been shown to be approximately 50–60% (17–22). In our population, 82% were still in treatment after 1 year, which is comparable (80% or higher) to regular opioid agonist clinics in Sweden (30). Thus, we draw the conclusion that SEPs may be feasible for treatment referral even with respect to longer course in treatment, and that they should be used as a link to treatment for people who inject drugs, as suggested also by other authors (36).

Our study has several limitations. One is that the relatively small total number of included subjects, which together with the high retention rate at 12 months prevented us from looking at variables potentially predicting retention. The setting being in Sweden, where all the included subjects were covered by the National Swedish Health Insurance, is another factor that might have influenced our retention rates and means that we cannot generalize our findings to settings where the cost for treatment could potentially influence retention rates. A strength, however, is that all the patients were referred to the same outpatient clinic with the same physician (the first author of this paper), which made follow-up easier and certain.

In summary, the present study was a 12-month follow-up of patients who had been rapidly transferred from a SEP to evidence-based treatment with methadone or buprenorphine/naloxone. In addition to previous data indicating that this may be an effective way of referral to treatment, we now also demonstrate that referral from SEP holds promise with respect to 12-month retention in treatment, demonstrating high retention comparable to that reported from more traditional and extensive intake procedures. This was found in a population with a high drug use severity on admission and no requirement for social stability. This highlights the importance of regarding SEPs not only as a harm reduction measure but also as a concrete possible link to effective evidence-based treatment.

## Ethics Statement

This study was carried out in accordance with the recommendations of the regional ethics board Lund, Sweden with written informed consent from all subjects in accordance with the Declaration of Helsinki. The protocol was approved by the regional ethics board Lund, Sweden.

## Author Contributions

MB: Ph.D. student in the project. Physician responsible for formal inclusion and evaluation of included patients. Principal writer of the manuscript. LE: did, as a medical student, preliminary statistical calculations and also took part in a preliminary draft of the manuscript. SN and PI: project assistant responsible for recruitment, randomization and assessment of patients, and provider of the studied intervention. Coworker in the follow-up of patients. KT: manager of the outpatient facility responsible for medical evaluation and treatment of included patients. Substantial activity in the practical setup of the study procedures and arrangements for medical evaluation and treatment. Coworker in the follow-up of patients and in randomization procedures. LB: co-supervisor of Ph.D. student MB. Scientific contributions related to manuscript writing and interpretations of results and limitations. AH: principal investigator of the project. Supervisor of Ph.D. student MB. Main responsible for study design including recruitment and randomization procedures, interventions, and scientific planning for the receiving treatment facility. All authors contributed to, approved of the manuscript, approved of submission to the journal.

## Conflict of Interest Statement

MB was invited to speak at the 7th Nordic Opioid Addiction Treatment Conference 2014, Helsinki, Finland. The conference was supported by RB Pharmaceuticals. MB received travel funds but no speaker fee. MB was also invited speaker at the Scottish Needle Exchange Conference 2014. The conference supported by Reckitt Benckiser, neo360, www.exchangesupplies.org, methameasure Ltd., pasante and solutions action management. MB did not receive any speaker fee but travel and accommodation was covered by the organizers. KT received travel funds from Nordic Drugs to attend ISAM in Malaysia 2014. According to Swedish legislation, her public employer paid for half of the amount. AH has no conflicts of interest to report related to the present study. He is involved in collaborations with two pharmaceutical companies in the preparation of clinical trials unrelated to the present paper and without receiving any personal salary from these bodies. He has been the local co-investigator in an epidemiological survey conducted by the independent research institute Research Triangle Institute (RTI), in a study for which RTI received funding from Shire, without any personal salary paid to the co-investigators. AH holds a position in addiction medicine as an employed researcher at Lund University. For this employment, the university receives funding from the national Swedish gaming operator (Svenska spel) owned by the Swedish government, as part of the company’s legislation-based responsibility for prevention and research against gambling problems. The other authors report no relevant financial conflicts.
